# Hyaluronic Acid-Coated Bovine Milk Exosomes for Achieving Tumor-Specific Intracellular Delivery of miRNA-204

**DOI:** 10.3390/cells11193065

**Published:** 2022-09-29

**Authors:** Dan Li, Liang Gong, Han Lin, Surui Yao, Yuan Yin, Zhifang Zhou, Jie Shi, Zhimeng Wu, Zhaohui Huang

**Affiliations:** 1 Wuxi Cancer Institute, Affiliated Hospital of Jiangnan University, Wuxi 214122, China; 2 Key Laboratory of Carbohydrate Chemistry & Biotechnology, Ministry of Education, School of Biotechnology, Jiangnan University, Wuxi 214122, China

**Keywords:** hyaluronic acid, milk exosomes, tumor-specific delivery, CD44, microRNA

## Abstract

Cell type-specific drug delivery is a straightforward strategy to achieve targeted cancer therapy and reduce side effects. Hyaluronic acid (HA), an U.S. Food and Drug Administration (FDA)-approved biocompatible carbohydrate polymer, has been extensively employed as a targeting ligand for a drug delivery system due to its natural ability to bind to tumor cells overexpressing cluster of differentiation 44 (CD44) receptors. Here, we report the preparation and antitumor efficacy of HA-coated bovine milk exosomes (HA-mExo) for tumor-specific delivery of microRNA-204-5p mimics (miR-204). The exosome-based delivery formulation was prepared with miR-204 encapsulated inside the lumen and HA displayed outside the membrane. The resultant formulation of HA-mExo-miR204 was able to specifically target CD44-positive cancer cells, with a concomitant increase in the intracellular uptake of miR-204. Compared to the uncoated mExo-miR204 formulation, HA-mExo-miR204 showed significantly increased antitumor efficacy both in vitro and in vivo. Importantly, HA-mExo-miR204 showed excellent biocompatibility and did not cause significant systemic toxicity. Given that both HA and bovine milk exosomes are low-cost and highly accessible biogenic materials with broad biomedical applications, HA-decorated bovine milk exosomes can be proven to be a practical drug delivery system of RNA drugs for targeted cancer therapy.

## 1. Introduction

Exosomes are a subpopulation of naturally secreted extracellular vesicles that have the endosomal origin and features of nanoscale diameters ranging from 40 to 160 nm (100 nm on average) [1]. Exosomes naturally carry biomolecule cargos of proteins, nucleic acids, and lipids, which can be transported from host cells to recipient cells for intercellular communication [2,3]. In light of this natural ability, exosomes have been extensively explored as drug delivery systems for therapeutic agents, including chemotherapy drugs and biological drugs for cancer therapy [4,5,6,7]. Although exosomes can be isolated from all types of cultured cells, bovine milk represents an ideal source of exosomes in terms of easy manipulation, low cost, and the potential to scale-up production [8,9]. The isolation of exosomes with high yield and quality has been a challenging process, and several isolation methods have been described for laboratorial use, such as ultracentrifugation, size exclusion chromatography, filtration, isoelectric precipitation, and polymer-based precipitation [9]. Taking into consideration the inexpensive milk resources and techniques, ultracentrifugation is the most feasible approach for the industrial preparation of milk exosomes. In addition, bovine milk exosomes (mExo) were shown to have inherent advantages of being safe, biocompatible, and less immunogenic [10]. Numerous studies have demonstrated the excellent prospects of milk exosomes as drug delivery tools for cancer therapy [11,12]. As natural delivery carriers, exosomes have been shown to have broad biodistribution in vivo, and thus, modifications of the membrane surface with target-specific ligands are often required to achieve cell type-specific delivery of drug cargos for targeted cancer therapy [13,14]. We have recently reported the tumor-specific delivery of doxorubicin (Dox) through mExo with hyaluronic acid (HA) decoration, due to HA’s ability to specifically bind to the cluster of differentiation 44 (CD44), which is a well-recognized therapeutic marker overexpressed in specific tumor cells [15,16]. HA is an FDA-approved carbohydrate polymer with excellent biocompatibility and safety, which makes it an ideal tumor-targeting ligand for developing target-specific drug delivery systems [17,18]. It was also shown that cancer cells with CD44 overexpression were more efficient than normal cells to internalize HA, supporting the feasibility of tumor-specific drug delivery employing HA as the targeting ligand [19]. This rational combination of biogenic materials is a practical solution to expand the capacity of exosome-based drug delivery systems.

Low intracellular delivery efficiency is one of the significant obstacles on the path to developing RNA-based drugs, which commonly show poor stability, limited intracellular uptake, and non-cell type specificity [20,21]. MicroRNA (miRNA) is a family of short noncoding RNAs that naturally exist in all cells. Due to their inherent ability to regulate specific target genes, mimics of natural miRNAs have been emerging as promising drug candidates for cancer therapy [22,23]. We and others have demonstrated that miRNA-204-5p (miR-204) was able to inhibit tumor growth, metastasis, and chemoresistance, highlighting it as a promising pan-cancer suppressor [24,25,26,27,28,29,30]. As miRNA affects its targets through genetic regulations inside the cells, efficient intracellular delivery is the prerequisite for developing miRNA drugs. Drug delivery with nano-carriers is a straightforward strategy for miRNA drugs to avoid nuclease-mediated degradation and enhance the intracellular uptake efficiency by the direct encapsulation of miRNA through natural and unnatural nano-systems, such as liposomes, exosomes, polymers, and other nanoparticles [7,28,29]. Due to their inherent and unique properties, exosomes showed apparent advantages in the delivery of nucleic acid drugs [7]. We previously attempted to utilize cell-derived exosomes to deliver miR-204 for cancer therapy and found that exosome-encapsulated miR-204 was capable of sensitizing cancer cells to chemotherapy drugs in vitro and suppressed xenograft tumor growth in vivo [31]. However, it has to be noted that this formulation still requires cell-type specificity for achieving tumor-specific delivery and more efficient tumor therapy.

In this study, to further improve the antitumor efficacy of miR-204 through exosome-based drug delivery systems, we prepared HA-coated bovine mExos for achieving tumor-specific intracellular delivery of miR-204. The formulation of HA-mExo-miR204 was prepared with miR-204 encapsulated inside the lumen and HA displayed outside the membrane. HA-mExo-miR204 was able to specifically target CD44-positive cancer cells, with a concomitant increase in miR-204 intracellular uptake. Compared to the uncoated mExo-miR204 control formulation, HA-mExo-miR204 showed significantly increased antitumor efficacy both in vitro and in vivo. Importantly, HA-mExo-miR204 showed excellent biocompatibility and did not cause significant systemic toxicity. In addition to the biogenesis nature of HA and mExos, the easy and low-cost preparation and the excellent safety of this formulation are also significant advantages over other drug delivery carriers, and it can be proven to be a practical drug delivery system of miRNA drugs for targeted cancer therapy.

## 2. Materials and Methods

### 2.1. Materials

1,2-distearoyl-sn-glycero-3-phosphoethanolamine-N-[methoxy(polyethyleneglycol)-2000] (DSPE-PEG_2000_) was purchased from Avanti Polar Lipids, Inc., Birmingham, AL, USA. Hyaluronic acid (HA, 30 kDa) was purchased from QuFu GuangLong Biochem Co., Ltd. (Shandong, China). Fresh bovine milk was purchased from a local supermarket (500 mL, Brightdairy Co., Ltd., Shanghai, China). The miRNA-204 mimic conjugated with FAM fluorophore and random miRNA as a negative control (Supplementary Table S1) were synthesized by Sangon Biotech (Shanghai, China). Anti-RAB22A rabbit polyclonal antibody (IgG, 12125-1-AP) was purchased from Proteintech (Chicago, IL, USA). Anti-BCL2 mouse monoclonal antibody (IgG, sc-7382) was purchased from Santa Cruz Co., Ltd. (Santa Cruz, CA, USA). Anti-β-actin rabbit polyclonal antibody (IgG, D110001-0025) was purchased from Sangon Biotech (Shanghai, China). HRP-conjugated goat anti-rabbit antibodies (IgG, A0208) and HRP-conjugated goat anti-mouse antibodies (IgG, A0216) were purchased from Beyotime Biotech (Shanghai, China). DSPE-PEG_2000_-HA was prepared as we described previously [15].

### 2.2. Cell Culture and Animals

Human breast cancer cell lines, including MDA-MB-231 and MCF-7, and human embryonic kidney cell line HEK293 were cultured in Dulbecco’s modified Eagle’s medium (DMEM, Corning Inc., 10-013-CVR, Corning, NY, USA) containing 10% fetal bovine serum (FBS, Tico, FBSEU500-500 mL) and 1% penicillin–streptomycin (Biosharp Co., Ltd., BL505A, Hefei, China) at 37 °C with 5% CO_2_. Female BALB/c nude mice (4–6 weeks old, 18–20 g) were obtained from Shanghai SLAC Laboratory Animal Co., Ltd. (Shanghai, China) and raised in specific pathogen-free (SPF) housing conditions. All animal operations were performed by following the guidelines listed in the Guide for the Care and Use of Laboratory Animals, and all animal experiments were approved by the Institutional Animal Care and Use Committee of Jiangnan University (JN.No20200710n1051020[165], Wuxi, China).

### 2.3. Exosomes Isolation, miR-204 Loading, and HA Coating

High-quality bovine milk exosomes were prepared as we previously reported [15]. Briefly, fresh bovine milk was first centrifugated at 13,000× *g* for 30 min to obtain skimmed milk, and the supernatant was further added with hydrochloride (HCl) to achieve pH 4.6 for the isoelectric precipitation of the caseins. A clear whey solution containing exosomes was obtained after the removal of the caseins by centrifugation at 13,000× *g* for 60 min. The pellet of exosomes was finally isolated by ultracentrifugation at 135,000× *g* for 60 min and then resuspended in PBS for storage and later use. The loading of the cargo miR-204 into the exosomes was achieved using an electroporation approach. Briefly, the exosomes were diluted to 4 × 10^12^ in 400 μL of Milli-Q. FAM fluorophore-labeled miR-204 was added to the above exosome solution for a final concentration of 400 nM. The mixture was then transferred into a prechilled 0.4-cm cuvette and electroporated at 350 V for a pulse time of 10 ms using a MicroPulser (Bio-Rad Laboratories Co., Ltd., Hercules, CA, USA) electroporation system. The mixture was kept on ice for at least 10 min before and after the electroporation pulse. Upon completion of the electroporation, the unencapsulated miR204 was then removed through a 10 kDa ultrafiltration filter by centrifugation at 3000× *g* for 10 min, and the buffer was exchanged for PBS. To determine the loading efficiency, a linear correlation standard curve of fluorescence intensity versus miR-204 concentration was generated (Figure S1). The miR-204 loaded into exosomes was determined accordingly, and the encapsulation efficiency was calculated by the following formula: miR-204 encapsulation efficiency (%) = (calculated miR-204 in exosomes/the total amount of miR-204) × 100%. Finally, the HA coating of the miR-204-loaded exosomes was achieved by incubating DSPE-PEG_2000_-HA (40 μg) with the miR-204-loaded exosomes (4 × 10^12^) for 48 h at 37 °C, followed by low-speed centrifugation (4000× *g*) to remove excess HA derivatives.

### 2.4. Size, Zeta Potential, Concentration, and Morphology Characterization of HA-mExo-miR-204

The particle size and zeta potential of the HA-mExo-miR204 were determined by dynamic light scattering using Zetasizer and Nano-ZS (Malvern Panalytical Ltd., Malvern, UK). The nanoparticle concentrations of mExos and HA-mExo-miR204 were determined by nanoparticle tracking analysis using Nanosight (Malvern Panalytical Ltd., Malvern, UK). The morphology of mExos and HA-mExo-miR-204 was recorded using a transmission electron microscope (TEM) (H-7650, HITACHI, Tokyo, Japan) at 100 kV after staining with 2% phosphotungstic acid.

### 2.5. Western Blot

Cells were seeded at a density of 2 × 10^6^ cells per well in 6-well plates and cultured for 12 h. After the treatments as described in the corresponding text, the cells were incubated with RIPA lysis buffer on ice for 30 min, and the cell lysate was centrifuged at 12,830× *g* for 5 min to remove cell debris. The supernatant proteins were collected and boiled in a sample loading buffer at 99 °C for 10 min. The samples were then separated by 10% SDS-polyacrylamide gel electrophoresis (SDS-PAGE) at 120 V for 1 h and subsequently transferred from the gel to a polyvinylidene fluoride (PVDF) membrane. The membrane was blocked with 5% non-fat milk in tris-buffered saline-tween 20 buffer (TBST) (Tris 1 M, NaCl 5 M, and Tween-20 0.05%, pH 8.0) at 37 °C for 30 min, followed by incubation with the indicated primary antibodies overnight at 4 °C. After washing three times with TBST, the membrane was further incubated with HRP-labeled secondary antibody (diluted in a blocking buffer) on a shaker at 37 °C for 1 h. After washing three times with TBST at 5-min intervals, an HPR-catalyzed signal was developed using an ECL Western blotting kit. The images were recorded using a ChemiDocXRS imager system (Bio-Rad Laboratories Co., Ltd., Hercules, CA, USA).

### 2.6. In Vitro Delivery Specificity and Efficiency of HA-mExo-miR204

For the immunocytochemistry analysis of the delivery specificity of HA-mExo-miR204, all cells were seeded at a density of 4 × 10^4^ cells per well in 24-well plates and cultured for 12 h. Cells were incubated with mExo-miR204 as a control or HA-mExo-miR204 for a final concentration of 100 nM of miR-204. PBS was used as a negative control. After incubation for 2 h, the cells were first fixed with 4% paraformaldehyde for 15 min and washed three times with PBS. Then, the cells were stained with 5 μg/mL of 4′,6-diamidino-2-phenylindole (DAPI) for 10 min and washed three times with PBS. Finally, cell staining was observed and imaged under a fluorescence microscope (Olympus Co., Ltd., CKX53, Tokyo, Japan). For the quantitative determination of the delivery efficiency, all cells were seeded at a density of 2 × 10^3^ cells per well in 96-well plates and cultured for 12 h. Subsequently, the cells were treated with mExo-miR204 or HA-mExo-miR204 for a final concentration of 100 nM of miR-204. Finally, after washing with PBS, each well was added with 100 μL PBS for fluorescent signal reading. The fluorescent intensity was recorded using a microplate reader (Synergy H4, Bio-Tek Instruments, Inc., Winooski, VT, USA) at 490 nm (excitation) and 520 nm (emission).

### 2.7. In Vitro Safety of HA-mExos

For the cytotoxicity assay, the effect of HA-mExos on cell viability was tested using a CCK-8 assay. Briefly, the cells were seeded at a density of 5 × 10^3^ cells per well in 96-well plates and cultured for 12 h. Subsequently, the medium was replaced with fresh complete medium in the presence of DSPE-PEG_2000_-HA, mExos, or HA-mExos. After incubation for 48 h, the cells were washed with PBS and further incubated with 100 μL of fresh medium containing 10% CCK-8 reagent for 2 h. The absorbance was recorded using a microplate reader (iMark, Bio-Rad, Hercules, CA, USA) at 450 nm.

For the hemocompatibility of HA-mExos in vitro, a hemolysis assay was performed. Briefly, the blood of BALB/c nude mice was collected with an anticoagulant to obtain red blood cells (RBCs). Subsequently, 300 μL erythrocyte suspension (2%, in saline) was mixed with 300 μL mExos or HA-mExos at different concentrations, and incubated for 2 h at 37 °C. Following centrifugation at 2500× *g* for 10 min, the absorbance of the supernatant was measured at 540 nm using a microplate reader. Erythrocytes treated with ultrapure water were set as the positive control, and erythrocytes treated with saline were set as the negative control. The hemolysis ratio (%) was calculated using the following formula.

Hemolysis ratio (%) = (A sample-A negative control)/(A positive control − A negative control) × 100%

A hemolysis ratio of less than 5% is regarded as nontoxic. The morphology of precipitated erythrocytes was also observed under a light microscope to further check the effect of these formulations on the integrity of the erythrocyte.

### 2.8. Cell Proliferation Assay

The effect of HA-mExo-miR204 on cancer cell proliferation was determined using a CCK-8 cell viability assay. Briefly, cells were seeded at a density of 2 × 10^3^ cells per well in 96-well plates and cultured for 12 h. Subsequently, the cells were incubated with mExo-miR204 or HA-mExo-miR204 for a final concentration of 100 nM of miR-204, and PBS was used as a negative control. After incubation for 6 h, the cells were further cultured in fresh medium for 72 h. Finally, the cell viability was determined as described in Section 2.7.

### 2.9. Colony Formation Assay

The effect of HA-mExo-miR204 on the proliferation of single tumorigenic cells was determined by colony formation assays. Cells were seeded at a density of 500 cells per well in 6-well plates and cultured for 12 h. Subsequently, mExo-miR204 or HA-mExo-miR204 were added to the cell medium to achieve the final concentration of 50 nM of miR-204, and the cells were further cultured for 8 days. Formed cell colonies were first fixed with 4% paraformaldehyde for 15 min and washed three times with PBS. Then the colonies were stained with 0.5% crystal violet for 15 min. After washing five times with PBS, the number of colonies from each well was counted.

### 2.10. In Vivo Antitumor Efficacy

The in vivo antitumor efficacy of HA-mExo-miR204 was evaluated in an MDA-MB-231 cell line-derived xenograft tumor mouse model. To establish the xenograft tumor model, female BALB/c nude mice were implanted with 5 × 10^6^ cells (MDA-MB-231 cells) subcutaneously in the right flank. After 10 days, tumor-bearing mice were randomly divided into 3 groups (*n* = 5–7). The grouped mice were intravenously injected with PBS (control), mExo-miR204 (0.2 nmol miR-204 per dose), or HA-mExo-miR204 (0.2 nmol miR-204 per dose) every 2 days 6 times. The body weights and tumor volumes of the mice were measured every 2 days. The tumor volume was calculated as indicated below: V = 1/2 × length × width^2^. On day 12, the mice were sacrificed and the tumors were collected for tumor weight analysis.

### 2.11. In Vivo Distribution of HA-mExo

The in vivo distribution of HA-mExo was visualized by labeling the exosomes with a lipophilic fluorophore 1,1-dioctadecyl-3,3,3,3-tetrametylindotricarbocyanine iodide (DiR). Briefly, mExo or HA-mExo (1 × 10^11^) was mixed with 2.5 mg DiR, and the mixture was incubated at 37 °C for 30 min. The unbound DiR was removed using a 30 kDa ultrafiltration filter by centrifugation at 3000× *g* for 30 min. Female BALB/c nude mice were administered with a single *i.v*. dose of mExo (2 × 10^10^) or HA-mExo (2 × 10^10^). After 24 h, the major organs of the mice were collected at the time of euthanasia, and images were recorded using an in Vivo Fluorescence Imaging System (IVIS SPECTRUM, PerkinElmer, Waltham, MA, USA).

### 2.12. Histological Analysis of Antitumor Effect and Systemic Toxicity of HA-mExo-miR204

To further assess the antitumor effect and the potential systemic toxicity of HA-mExo-miR204, tumors and major organs of the mice from in vivo antitumor experiments were excised for histological analysis. Sections of tumor, heart, liver, spleen, lung, and kidney were stained with hematoxylin and eosin for the assessment of morphological changes, and images were recorded using an electronic microscope.

### 2.13. Immunohistochemistry

The effect of HA-mExo-miR204 on the expressions of RAB22A and BCL2 in tumor tissue was determined by immunohistochemistry analysis. Briefly, the rehydrated slides of tumor tissues were repaired by 0.01 M citrate buffer (pH 6.0) for 10 min in boiling water. After cooling down to room temperature naturally and blocked with goat serum, the slides were further incubated with the anti-RAB22A or BCL2 monoclonal antibody (1:50 dilution) at 4 °C overnight. The slides were then incubated with HRP-conjugated goat anti-rabbit antibodies or HRP-conjugated goat anti-mouse antibodies and stained with DAB. Finally, the slides were stained with hematoxylin, dehydrated in different concentrations of ethanol, and mounted using gum. Images were recorded using an electronic microscope.

### 2.14. Statistical Analysis

All data were presented as the mean ± standard deviation (SD) of at least three parallel experiments. The statistical analyses were analyzed using Student’s *t*-test. Statistical significance was set as follows: * *p* < 0.05, ** *p* < 0.01, *** *p* < 0.001, **** *p* < 0.0001, N.S., not significant.

## 3. Results

### 3.1. Preparation and Characterization of HA-mExo-miR204

**Figure 1:**
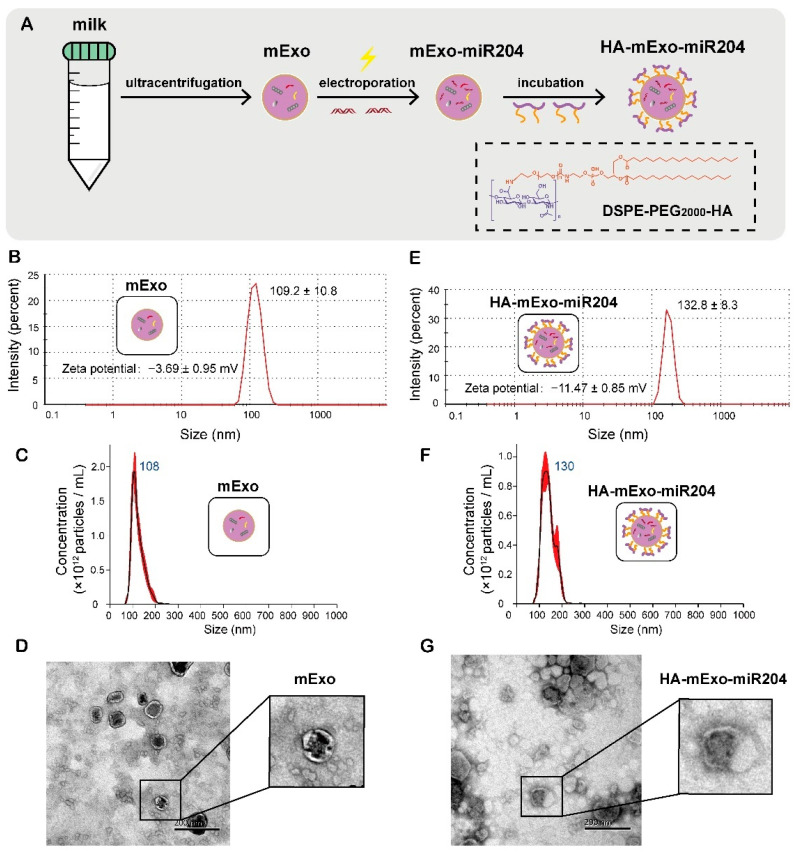
Preparation and characterization of HA-mExo-miR204. (**A**) Schematics of preparation of HA-decorated bovine mExos containing miR-204 (HA-mExo-miR204). Hydrodynamic size and zeta potential of the intact mExos (**B**,**E**) HA-mExo-miR204 were determined by DLS analyses. Concentration and size distribution of the intact mExos (**C**) and HA-mExo-miR204 (**F**) were determined by NTA analyses. TEM images of the intact mExos and HA-mExo-miR204 were shown in (**D**,**G**), respectively. Scale bar: 100 nm.

The HA-coated bovine mExos containing miR-204 was prepared by following the procedures as illustrated in Figure 1A. High-quality mExo was isolated from bovine milk as we described previously [15]. The loading of miR-204 into mExos was achieved through an electroporation approach, and the encapsulation rate of miR-204 was calculated as 98% through a standard curve of miR-204 (Figure S1). The surface of cargo-loaded mExos was further coated with the membrane-insertable HA derivative of DSPE-PEG_2000_-HA through its hydrophobic lipid tail, and the resultant formulation was termed HA-mExo-miR204 for the sake of brevity (Figure 1A). We chose 30-kDa HA for the modification because it showed excellent affinity for CD44 among other HA molecular weight variants [32]. DSPE-PEG is an amphiphilic molecule that has been approved by the FDA for medical applications and has also been demonstrated in a case of microvesicle modification for tumor-targeting [33]. HA-mExo-miR204 was first characterized by dynamic light scattering (DLS) analyses and the average hydrodynamic size was determined as 132.8 ± 8.3 nm, which was larger than that of the intact mExos (109.2 ± 10.8 nm) (Figure 1B,E). DLS analyses also showed that the zeta potential of HA-mExo-miR204 and the intact mExos was -11.47 ± 0.85 mV and -3.69 ± 0.95 mV, respectively (Figure 1B,E). The size of these formulations was confirmed by nanoparticle tracking analysis (NTA), and the results are generally consistent with the results obtained from DLS analyses (Figure 1C,F). The morphology features of HA-mExo-miR204 were determined using transmission electron microscopy (TEM). The HA-mExo-miR204 kept the vesicular membrane structure as the intact mExos, suggesting that inserting the DSPE-PEG_2000_-HA did not cause detectable changes to the membrane integrity of the mExos (Figure 1D,G). To further confirm the encapsulation of miR-204, HA-mExo-miR204 was analyzed by agarose gel electrophoresis. As shown in Figure S2, the unencapsulated miR-204 was found to migrate into the lower molecular weight range in the gel, whereas miR-204 from HA-mExo-miR204 lost mobility and was retained in the loading wells, indicating that the miR-204 was successfully encapsulated into the mExos. Moreover, we determined the storage stability of the HA-mExo-miR204. After storage at −20 °C for up to 7 days, the formulation was shown to be relatively stable, as no significant changes were detected in either DLS or agarose gel electrophoresis analyses (Figure S3).

### 3.2. HA-mExo-miR204 Showed Increased Delivery Specificity and Efficiency

**Figure 2:**
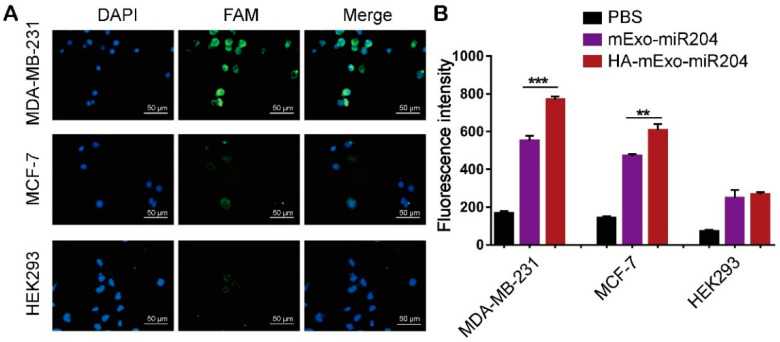
In vitro delivery specificity and efficiency of HA-mExo-miR204. (**A**) Immunocytochemistry analyses of miR-204 cellular uptake. Cancer cells were incubated with HA-mExo-miR204 for 2 h. Blue: nucleus stained by DAPI; green: FAM fluorophore-labeled miR-204. Scale bars: 20 μm. (**B**) Quantitative analyses of miR-204 cellular uptake by determining the fluorescent signals inside the cells. ** *p* < 0.01, *** *p* < 0.001.

We next determined the delivery specificity and efficiency of HA-mExo-miR204. To this end, we first searched in the Cancer Cell Line Encyclopedia (CCLE) for cancer cells with a high expression level of CD44 mRNA. Two breast cancer cell lines, i.e., MDA-MB-231 and MCF-7, were chosen, as they showed different expression levels of CD44 mRNA (Figure S4A). The CD44 protein expression in these cells was also confirmed by Western blotting (Figure S4B). The cellular uptake specificity of HA-mExo-miR204 was assessed by tracking the FAM fluorophore-labeled miR-204 using an immunocytochemistry assay. As shown in Figure 2A, after incubation with HA-mExo-miR204 for 2 h, a high intensity of green fluorescence (miR-204) was detected in MDA-MB-231 cells and it was less intensive in MCF-7 cells, in which the CD44 expression was significantly lower than that in MDA-MB-231 cells. Moreover, a very limited amount of miR-204 uptake was detected in the negative control cell line of HEK293, which was reported to hardly express CD44. We next compared the miR-204 delivery efficiency between the HA-mExo-miR204 and the uncoated control of mExo-miR204 in these cells using a quantitative assay. All cells were incubated with HA-mExo-miR204 or mExo-miR204 for 2 h, and the fluorescence intensity inside the cells was determined for quantifying the cellular uptake of miR-204, as its concentration was well proportional to the fluorescence intensity (Figure S1). As shown in Figure 2B, all cancer cells treated with HA-mExo-miR204 showed significantly higher fluorescence intensities than the same cells treated with mExo-miR204, while no significant difference was found between these two formulations in the HEK293 cells. Together, these results suggest that HA decoration enabled the mExo to deliver miR-204 to target cancer cells with enhanced specificity and efficiency.

### 3.3. HA-mExo-miR204 Showed Increased In Vitro Antitumor Activity

**Figure 3:**
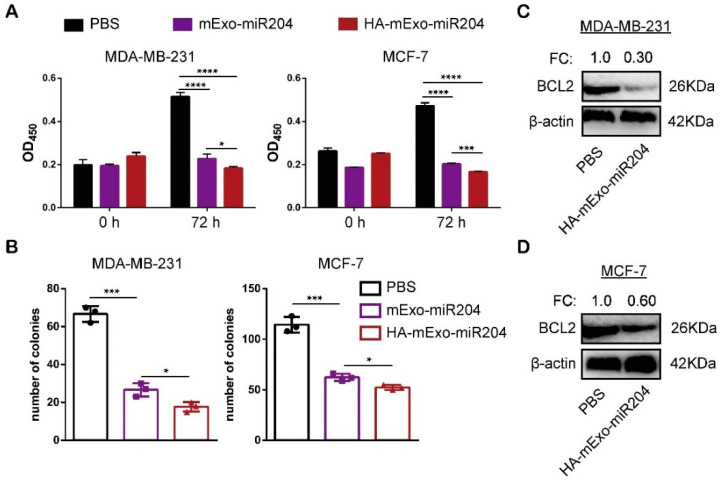
In vitro antitumor activity of HA-mExo-miR204. (**A**) Cancer cells were incubated with mExo-miR204 or HA-mExo-miR204 for 6 h, and the anti-proliferation effect was determined by CCK-8 assays. (**B**) Cells were grown at a low density for 8 days and the anti-proliferation effect of mExo-miR204 and HA-mExo-miR204 on single tumorigenic cells was determined by a colony formation assay. MDA-MB-231 (**C**) and MCF-7 (**D**) cells were treated with PBS or HA-mExo-miR204 for 12 h and the expressions of BCL2 and RAB22A were determined by Western blotting. * *p* < 0.05, *** *p* < 0.001, **** *p* < 0.0001.

We further tested whether HA-mExo-miR204 was able to improve the in vitro antitumor activity of miR-204. The effects of HA-mExo-miR204 and mExo-miR204 on cancer cell proliferation were compared using the CCK8 cell viability assay. As shown in Figure 3A, both the treatments of mExo-miR204 and HA-mExo-miR204 potently suppressed the proliferation of all cancer cell lines compared to the PBS negative control. Notably, among all the cancer cell lines tested, HA-mExo-miR204 was significantly more potent than mExo-miR204 in inhibiting cell proliferation, which can be attributed to the increased intracellular delivery of miR-204 through HA-coated exosomes (Figure 2B). The effect of these formulations on the proliferation of a single tumorigenic cell was determined by a colony formation assay. Both formulations of HA-mExo-miR204 and mExo-miR204 robustly reduced the colony formation ability of all cancer cells, while HA-mExo-miR204 was again more potent than mExo-miR204 in this assay (Figure 3B). We previously reported that miR-204 could suppress tumor cell proliferation by down-regulating multiple targets, such as BCL2 and RAB22A [27,28,29]. Thus, we further determined the effect of HA-mExo-miR204 on the expression of BCL2 and RAB22A in these cancer cells. Western blotting analyses showed that the expressions of BCL2 and RAB22A in MDA-MB-231 cells were significantly reduced by HA-mExo-miR204 treatment (Figure 3C and Figure S5). The reduced expression of BCL2 associated with HA-mExo-miR204 treatment was also detected in MCF-7 cells (Figure 3D). In addition, we tested whether HA-mExo-miR204 could increase the chemosensitivity of Dox against these cancer cells. As shown in Figure S6, a significant synergistic effect was seen in MCF-7 cells treated by Dox and HA-mExo-miR204 together, compared to the same cells treated by Dox only. However, the synergistic effect was not found in MDA-MB-231 cells. This variation in synergistic effects might be associated with the different mechanisms involved in the Dox-resistance for different types of tumor cells, which would be interesting to explore in a future study [34].

### 3.4. HA-mExo-miR204 Showed Potent In Vivo Antitumor Efficacy

**Figure 4:**
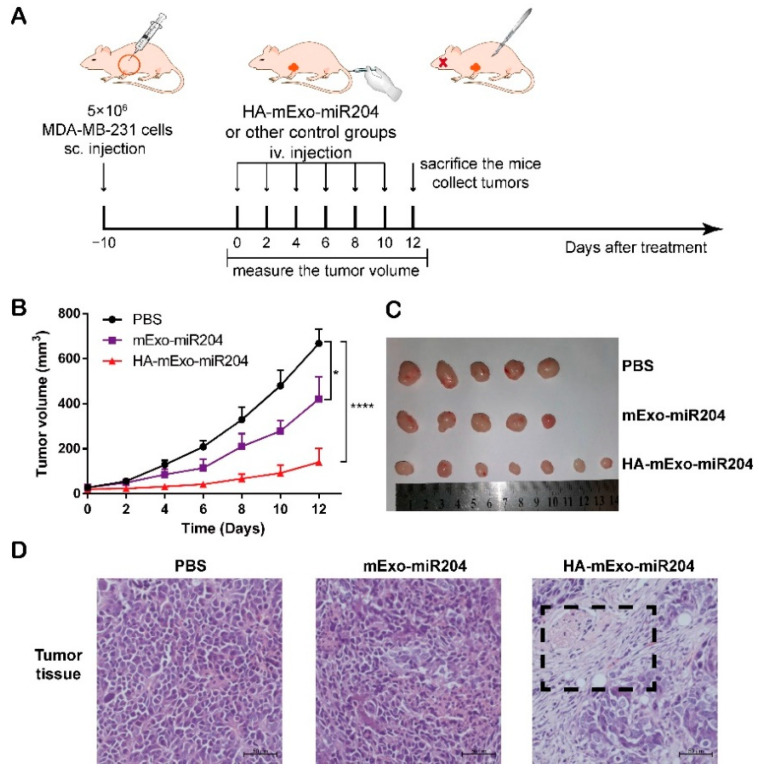
In vivo antitumor efficacy of HA-mExo-miR204. (**A**) Schematics of mice model construction and therapeutic schedule. (**B**) Average tumor volume of mice during the therapeutic course. (**C**) Tumor images of mice after the therapy. (**D**) Histological analysis of tumor sections with HE staining. The region showing significant apoptosis was highlighted by the black dashed frame. Scale bars: 50 μm. * *p* < 0.05, **** *p* < 0.0001.

**Figure 5:**
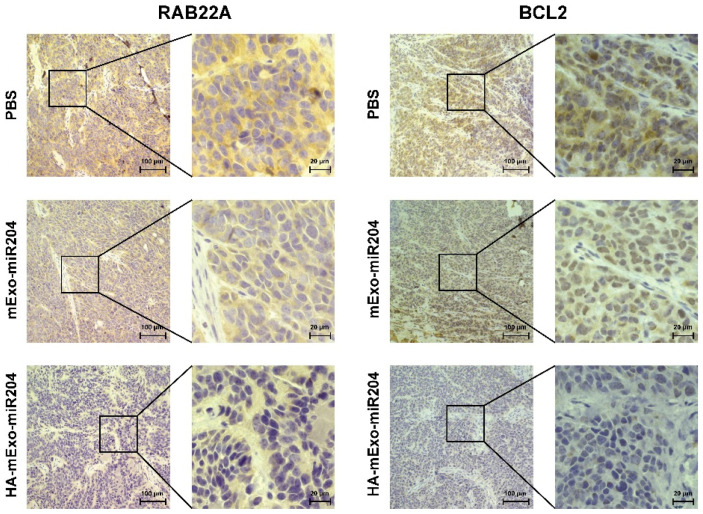
IHC analysis of RAB22A and BCL2 protein expressions in xenograft tumor. Sections of tumor tissues from mice were stained with RAB22A and BCL2 antibodies. Black boxed regions are enlarged in the right images. Scale bars: 100 μm (left); 20 μm (right).

The in vivo therapeutic effect of HA-mExo-miR204 was assessed in a nude mouse model bearing xenograft tumors derived from MDA-MB-231 cells. The tumor-bearing mice were intravenously administered with PBS, mExo-miR204, or HA-mExo-miR204 (0.2 nmol miR-204 per dose) every 2 days 6 times (Figure 4A). Compared to the PBS-treated group, the mExo-miR204 treatment only caused a moderate reduction in tumor growth, with an endpoint tumor growth inhibition ratio of 32% (Figure 4B,C and Figure S7B). However, HA-mExo-miR204 showed remarkably improved antitumor efficacy, as shown in the evidence that the xenograft tumor growth was dramatically slowed down (Figure 4B,C) and the endpoint tumor growth inhibition ratio was ca. 69% (Figure S7B). Notably, during the entire therapeutic course, all the treatments did not cause a significant reduction in the mice’s body weight (Figure S7A). Moreover, the antitumor efficacy of HA-mExo-miR204 was confirmed through histological analyses of the endpoint tumor tissues with HE staining. Only moderate apoptosis occurred in the tumor tissues of mice treated with mExo-miR204 and significantly increased apoptotic or lytic cancer cells that existed in the tumor tissues of mice treated with HA-mExo-miR204, especially inside the region highlighted by the black dashed frame (Figure 4D). In addition, IHC analyses showed that the expressions of RAB22A and BCL2 were slightly down-regulated in tumors from mExo-miR204-treated mice compared to the control mice, and a more potent reduction of that was seen in the tumors from HA-mExo-miR204-treated mice (Figure 5).

### 3.5. Safety Evaluation of HA-mExo-miR20

**Figure 6:**
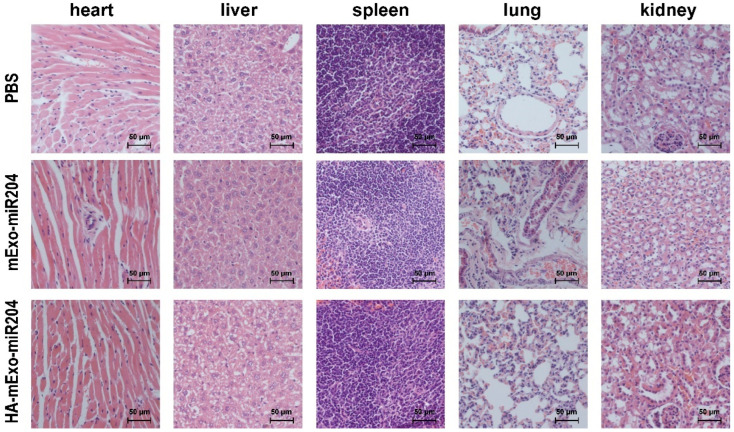
Histological analyses of major organs of mice treated with HA-mExo-miR204. Sections of heart, liver, spleen, lung, and kidney were stained with hematoxylin/eosin. Scale bars: 50 μm.

Finally, we assessed the safety of HA-mExo-miR204. For cytocompatibility, MDA-MB-231, MCF-7, and HEK293 cells were incubated with DSPE-PEG_2000_-HA (10 μg), intact mExos (1 × 10^12^), and HA-mExos (1 × 10^12^), respectively. For up to 48 h, all the treated cells retained a cell viability similar to that of the untreated cells (Figure S8), indicating that HA-mExos had no significant cytotoxicity. For blood compatibility, red blood cells (RBCs) were treated with the intact mExos and HA-mExos at concentrations of 2.5 × 10^12^ particles/mL for 3 h. The results show that both the intact mExos and HA-mExos had no detectable influence on the integrity of the RBC membranes, and the hemolysis ratios of the intact mExos and HA-mExos were both less than 5% (Figure S9). For in vivo safety, we first traced the distribution of HA-mExo and intact mExo in mice by DiR labeling (Figure S10). We found significant accumulation of the intact mExo in the liver and lung, as reported by others [35,36], but this accumulation pattern became less intensive for HA-mExo, which might be associated with the altered interactions of exosomes with cells caused by the surface ligand coating [37]. We further checked whether this formulation caused systemic toxicity during the therapy course. Histopathological analyses with HE staining revealed that neither HA-mExo-miR204 nor mExo-miR204 induced tissue or cellular damage in the heart, liver, spleen, lung, or kidney (Figure 6), therefore indicating that these formulations are generally safe and do not cause any obvious systemic toxicity.

## 4. Discussion

In this study, HA was employed as a tumor-targeting ligand to decorate and functionalize bovine mExos for achieving tumor-specific intracellular delivery of miR-204. As expected, the resultant formulation of HA-mExo-miR204 showed significantly increased antitumor efficacy both in vitro and in vivo.

HA is an FDA-approved carbohydrate polymer that has broad medical applications. We chose HA as a targeting ligand to functionalize mExos because HA is biocompatible, biodegradable, noninflammatory, nonimmunogenic, and has low toxicity, in addition to its capability of tumor-specific binding through the CD44 receptor [38,39]. Our results confirm that both HA derivatives and HA-mExo showed excellent cytocompatibility and hemocompatibility in vitro. The entire HA-mExo-miR204 formulation was generally safe in mice, without causing significant systemic toxicity. Taking into account the similar biocompatibility of mExos, this rational combination of HA and mExos as a delivery tool should have excellent safety and prove to be a practical targeted-delivery system. In addition, we demonstrated that HA decoration was feasible to enable mExo to deliver miR-204 to target cancer cells with enhanced specificity and efficiency. However, it can be clearly observed that the uncoated control mExo-miR204 was also able to efficiently deliver the cargo, possibly by passive membrane fusion. This raises the concern that non-targeting delivery might also exist in the formulation of HA-mExo-miR204. Future studies should further clarify this issue.

Exosomes as natural nano-carriers have apparent advantages over traditional drug delivery materials, which well support the application of exosomes for developing a cell type-specific intracellular delivery system of miRNA [7,22]. For example, exosomes were shown to be more stable than synthetic liposomes in the body fluid environment [8]. Due to their biogenic nature, exosomes have excellent biocompatibility and high cellular uptake efficiency. In a previous attempt, we prepared cell-derived exosomes containing genetically overexpressed miRNA-204 as a delivery formulation [31]. Although significant antitumor activity of this formulation was observed, it has to be acknowledged that the preparation of this formulation was tedious and not cost-effective. The product might also have a low and heterogeneous encapsulation rate. To overcome these obstacles and make this formulation more practical, in the present contribution, we chose bovine milk exosomes for preparing the targeted delivery formulation in consideration of the exosome availability, easy manipulation, low cost, and potential to scale-up production [8,9]. In addition, we also used synthetic miRNA-204 to obtain a formulation with a high encapsulation rate, which can be achieved by more straightforward cargo-loading approaches, such as electroporation. These significant advantages of exosome preparation, miRNA loading, and surface modification are an important basis for preparing high-quality drug delivery formulations.

We have previously revealed that miR-204 was capable of down-regulating multiple targets, such as BCL2 and RAB22A, in tumor cells [24,25,26]. In this study, in the form of a drug delivery formulation, synthetic miR-204 was again confirmed to be able to inhibit tumor growth in vitro and in vivo, and the expressions of BCL2 and RAB22A were significantly down-regulated in tumor tissue. More importantly, we did not detect obvious toxicity of these formulations in mice, highlighting miR-204 as a promising and safe pan-cancer suppressor. In addition, HA-mExo-miR204 can selectively increase the chemosensitivity of Dox against specific cancer cells, indicating that miR-204 has the potential to overcome chemoresistance through a synergistic effect. Further, the antitumor potential of this synergistic effect and the mechanisms behind it should be studied in the future.

## 5. Conclusions

In summary, we demonstrated the great potential of HA-decorated mExo for the tumor-specific delivery of microRNA drugs. The formulation of HA-mExo-miR204 can specifically target CD44-positive tumor cells in vitro, with a concomitant increase in miR-204 intracellular uptake and anti-proliferation activity. HA-mExo-miR204 dramatically suppressed xenograft tumor growth in a mouse model, which was more potent than the non-targeting control of mExo-miR204. Importantly, HA-mExo-miR204 showed excellent compatibility in vitro and did not cause significant systemic toxicity in vivo, suggesting that this formulation is generally safe. Given that both the carbohydrate polymers of HA and mExo are low-cost and highly accessible materials that have been widely used for biomedical purposes, HA-decorated mExo can be proven to be a practical drug delivery system of microRNA for targeted cancer therapy.

## Supplementary Materials

The following supporting information can be downloaded at: https://www.mdpi.com/article/10.3390/cells11193065/s1, Table S1. List of miRNAs used in this study; Figure S1: Standard curve of miR-204 concentration versus the corresponding fluorescence intensity; Figure S2: Agarose gel electrophoresis of free miRNA-204 and mExo-miR204; Figure S3: Storage stability of HA-mExo-miR204; Figure S4: CD44 expression in cancer cells; Figure S5: Western blotting analysis of RAB22A expression; Figure S6: The effect of HA-mExo-miR204 on the chemosensitivity of Dox; Figure S7: Body weight and tumor weight of mice; Figure S8: Cytotoxicity of DSPE-PEG_2000_-HA, mExos and HA-mExos; Figure S9: In vitro hemocompatibility of mExos and HA-mExos; Figure S10: In vivo distribution of mExos and HA-mExos.
