# Supplementary material for: Hyaluronic Acid-Coated Bovine Milk Exosomes for Achieving Tumor-Specific Intracellular Delivery of miRNA-204
Source: Cells. 2022 Sep 29;11(19):3065. doi: 10.3390/cells11193065 (PMC9562169; doi:10.3390/cells11193065)
Supplement: Supplementary file 1 [file cells-11-03065-s001.zip › cells-1897163-supplementary.pdf]

cells

MDPI

Supplementary Material

Table S1. List of miRNAs used in this study.

Name	Sequences (5′ to 3′)	
miRNA-204 mimic (5′ FAM-tag)	Sense	5′-UUC CCU UUG UCA UCC UAU GCC U-3′
	Antisense	5′-A GGC AUA GGA UGA CAA AGG GAA-3′
random miRNA (Negative Control)	Sense	5′-UUC UCC GAA CGU GUC ACG UTT-3′
	Antisense	5′-ACG UGA CAC GUU CGG AGA ATT-3′

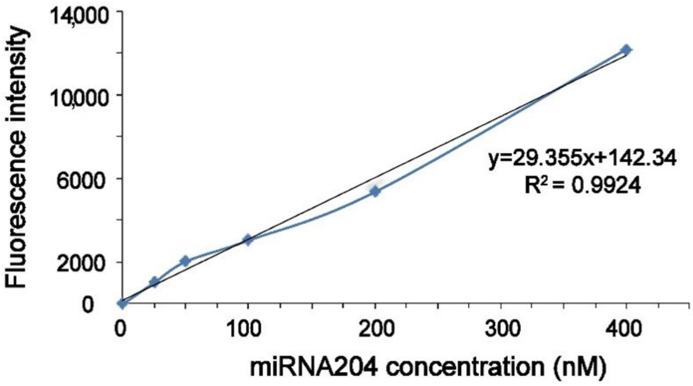

Figure S1. Standard curve of miR-204 concentration versus the corresponding fluorescence intensity.

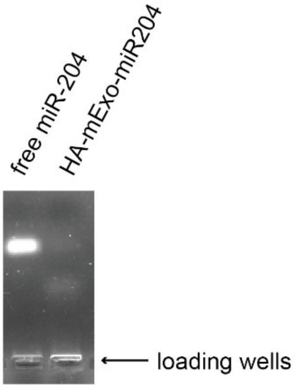

Figure S2. Agarose gel electrophoresis of free miRNA-204 and HA-mExo-miR204.

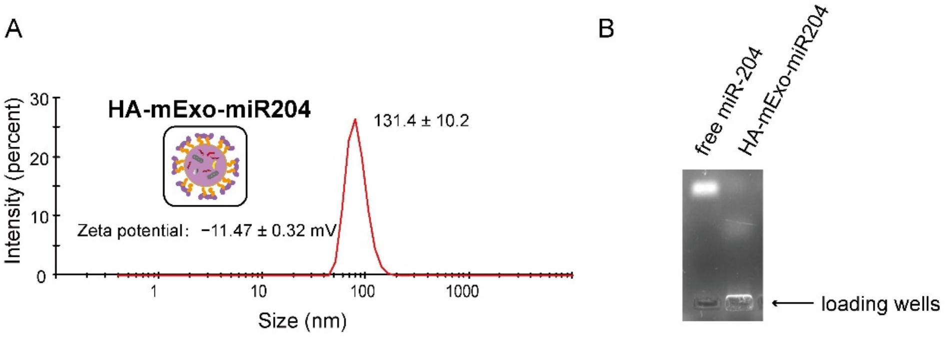

Figure S3. Storage stability of HA-mExo-miR204. DLS analysis (**A**) and agarose gel electrophoresis (**B**) of HA-mExo-miR204 after storage at –20 °C for 7 days.

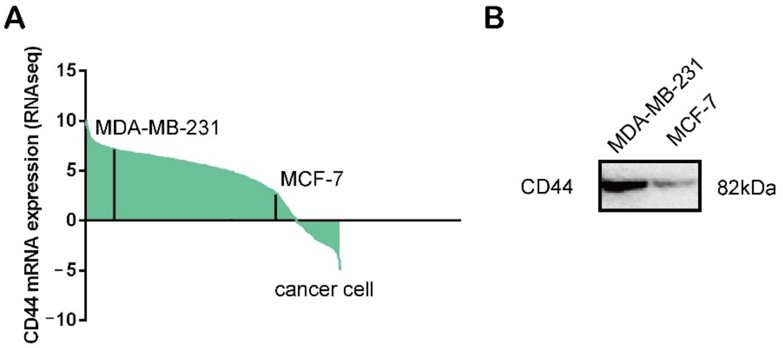

Figure S4. CD44 expression in cancer cells. (**A**) Relative expression of CD44 mRNA in MDA-MB-231 and MCF-7 cancer cell lines. (**B**) Western blot analysis of CD44 protein expression in MDA-MB-231 and MCF-7 cancer cell lines.

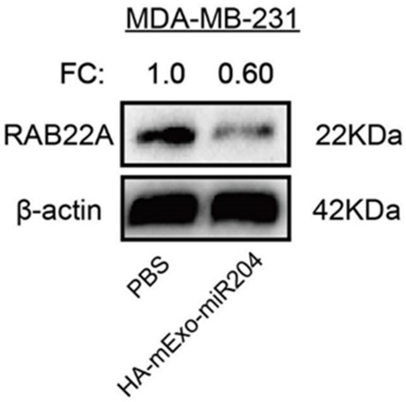

Figure S5. Western blotting analysis of RAB22A expression. MDA-MB-231 cells were treated with PBS or HA-mExo-miR204 for 12 h.

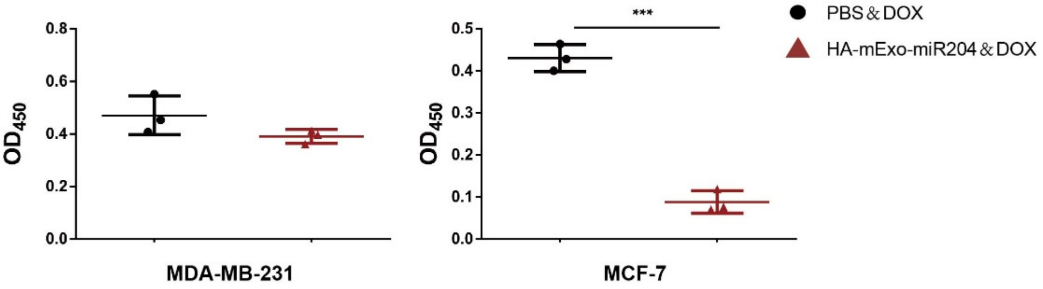

Figure S6. The effect of HA-mExo-miR204 on the chemosensitivity of Dox. Cancer cells were treated with PBS or HA-mExo-miR204 in combination with Dox for 12 h and the effect of HA-mExo-miR204 on Dox chemosensitivity was determined by CCK-8 cell viability assay. *** $p < 0.001$.

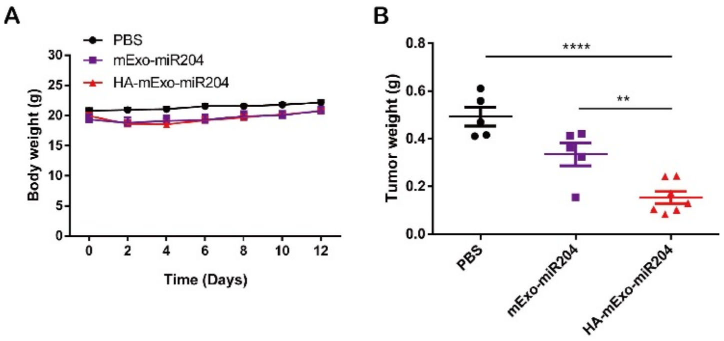

Figure S7. Body weight and tumor weight of mice. (**A**) Body-weight of tumor-bearing mice during the therapeutic course. (**B**) Endpoint tumor weight the mice after 12-days treatments. ** $p < 0.01$, **** $p < 0.0001$.

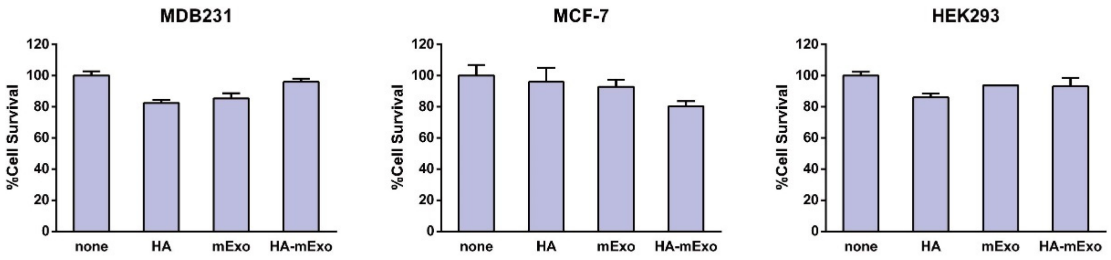

Figure S8. Cytotoxicity of DSPE-PEG2000-HA, mExos and HA-mExos. MDA-MB-231, MCF-7 and HEK293 cells were incubated with DSPE-PEG2000-HA, mExos or HA-mExos, respecively. After 48 h, cell viability was determined by CCK-8 assay.

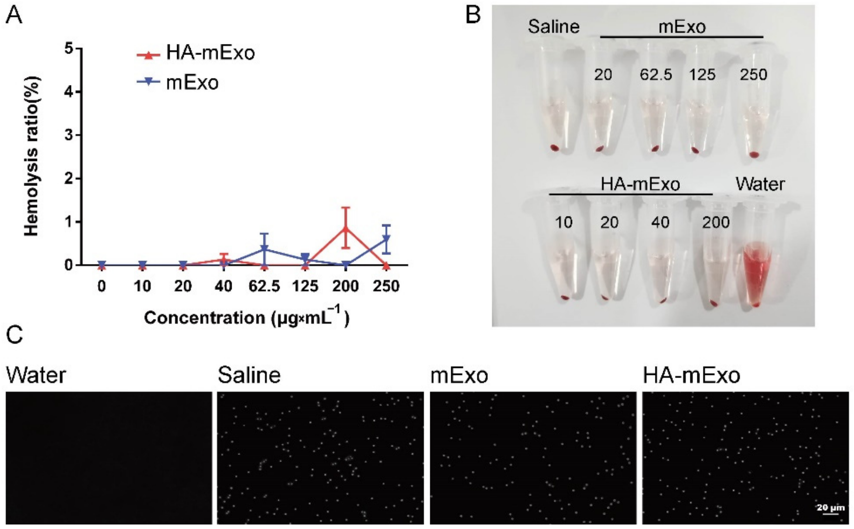

Figure S9. In vitro hemocompatibility of mExos and HA-mExos. (**A**) Hemolysis ratio of mExos and HA-mExos. (**B**) The images of red blood cells after incubation with mExos and HA-mExos. (**C**) Microscopy analysis of red blood cell membrane integrity after incubation with mExos and HA-mExos. Scale bars: 20 μm.

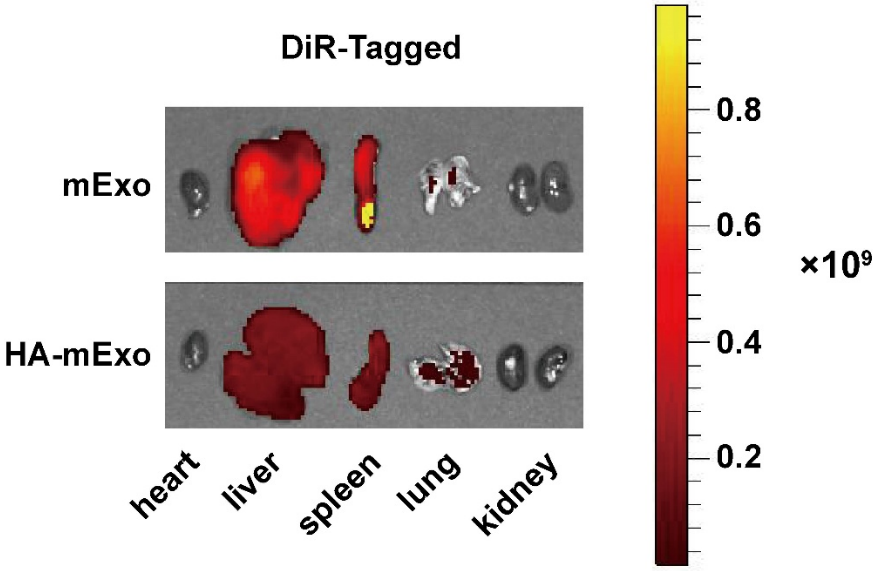

Figure S10. In vivo distribution of mExos and HA-mExos. The visualization of mExos and HA-mExos was achieved by labeling the exosomes with DiR dyes. Images of mice organs were recorded using an in vivo Fluorescence Imagine System (IVIS SPECTRUM, PerkinElmer, Waltham, MA, USA).
